# Predicting the Toxicity of Drug Molecules with Selecting Effective Descriptors Using a Binary Ant Colony Optimization (BACO) Feature Selection Approach

**DOI:** 10.3390/molecules30071548

**Published:** 2025-03-31

**Authors:** Yuanyuan Dan, Junhao Ruan, Zhenghua Zhu, Hualong Yu

**Affiliations:** 1 School of Environmental and Chemical Engineering, Jiangsu University of Science and Technology, Zhenjiang 212100, China; danyy@just.edu.cn (Y.D.); rjunhao@stu.just.edu.cn (J.R.); zhenghua_zhu@yeah.net (Z.Z.); 2 School of Computer, Jiangsu University of Science and Technology, Zhenjiang 212100, China

**Keywords:** toxicity prediction, molecule descriptors, quantitative structure–activity relationship (QSAR), feature selection, binary ant colony optimization, Tox21 challenge, Modred descriptor calculator, support vector machine

## Abstract

Predicting the toxicity of drug molecules using in silico quantitative structure–activity relationship (QSAR) approaches is very helpful for guiding safe drug development and accelerating the drug development procedure. The ongoing development of machine learning techniques has made this task easier and more accurate, but it still suffers negative effects from both the severely skewed distribution of active/inactive chemicals and relatively high-dimensional feature distribution. To simultaneously address both of these issues, a binary ant colony optimization feature selection algorithm, called BACO, is proposed in this study. Specifically, it divides the labeled drug molecules into a training set and a validation set multiple times; with each division, the ant colony seeks an optimal feature group that aims to maximize the weighted combination of three specific class imbalance performance metrics (F-measure, G-mean, and MCC) on the validation set. Then, after running all divisions, the frequency of each feature (descriptor) that emerges in the optimal feature groups is calculated and ranked in descending order. Only those high-frequency features are used to train a support vector machine (SVM) and construct the structure–activity relationship (SAR) prediction model. The experimental results for the 12 datasets in the Tox21 challenge, represented by the Modred descriptor calculator, show that the proposed BACO method significantly outperforms several traditional feature selection approaches that have been widely used in QSAR analysis. It only requires a few to a few dozen descriptors for most datasets to exhibit its best performance, which shows its effectiveness and potential application value in cheminformatics.

## 1. Introduction

During drug development, toxicity tests are generally required to guarantee patient safety [1,2]. Traditional toxicity tests are carried out in biochemistry laboratories; these are time-consuming and cause the deaths of many experimental animals [3]. In recent years, the in silico quantitative structure–activity relationship (QSAR) has been an effective and efficient alternative for predicting the toxicity of molecules produced during drug development [4,5,6]. It is noteworthy that, with the sustained accumulation of chemical structure–activity data and the rapid development of machine learning techniques, QSAR models are becoming increasingly accurate [7,8,9].

However, practical QSAR modeling processes usually face two challenges: a relatively high-dimensional feature (descriptor) distribution and a skewed distribution of active/inactive chemicals. These two data characteristics hinder the accuracy of the modeling for QSAR. In recent years, some feature selection techniques [10,11,12] and learning strategies focusing on imbalanced data [13,14,15] have been proposed. It is well known that feature selection removes irrelevant and redundant features in the original feature space, and the emerging feature selection algorithms can be roughly divided into one of three categories: filter [10,11], wrapper [12], and embedded [16]. Filter methods independently estimate the correlation strength of each feature with class labels and then rank them according to their scores to select those with the most differentiation for different classes. These methods are generally fast, but they fail to focus on the correlations among features. Wrapper approaches always select a feature subset and use a specific classifier to estimate its accuracy on a previously prepared validation set and then, according to the feedback, change the feature subset using some optimization strategies until an excellent feature subset is acquired that can produce an accurate enough result on the validation set. Since the classifier evaluation participates, wrapper methods are generally time-consuming, but the cooperative effect among features should be considered. As for the embedded feature selection, it is a niche strategy and is highly sensitive to the structure of underlying classifiers. That means that the features selected by a specific classifier may be useless for other classifiers. It is especially noteworthy that, in the context of QSAR, the existing feature selection approaches fail to effectively extract those features that can maximize the performance of the learning model in the context of skewed data distribution.

The aforementioned issue motivates us to design and develop new feature selection approaches in the context of imbalanced data distribution. In this study, a novel feature selection method based on the idea of ant colony optimization [17,18,19], called a binary ant colony optimization (BACO) algorithm, is proposed. Specifically, BACO arranges all features in order between the ant colony and the food source, and there are two paths from each feature to the next one. Here, one path denotes that the next feature should be selected, and the other one signals that the next feature is useless. For each ant, after it travels from the ant colony to the food source, a feature group can be extracted. Next, a support vector machine (SVM) classifier [20], which has been most widely used in various QSAR tasks [21,22,23,24], is trained based on the extracted feature group, and its quality is further evaluated according to the fitness function, which is a mixture of three different performance metrics: F-measure, G-mean, and MCC (Mathew correlation coefficient). These three metrics are specifically designed for evaluating the performance of the classification model on imbalanced data; thus, the fitness can accurately reflect the robustness of the feature group to skewed data distribution. In addition, to avoid producing overfitting and/or random results, the original training set can be divided multiple times into a sub-training set and a sub-validation set. For each sub-validation set, an optimum feature group can be obtained by conducting the BACO optimization procedure. Next, the frequency of each feature that emerges in all optimum feature groups is counted, and all features are ranked in descending order. Finally, some top-ranked features are extracted to train a new SVM classifier to classify instances in the testing set. Notably, BACO can be seen as a mixed-feature selection method, which first conducts a wrapper feature selection procedure and then uses the results to guide filter feature selection, aiming to uncover the features that are the most robust to skewed data distribution.

We use the proposed BACO algorithm to accomplish the toxicity prediction of drug molecules. Specifically, twelve datasets acquired from the Tox21 data challenge [25] are used to verify the effect of the BACO algorithm. Among the twelve datasets used, eight refer to the stress response (NR), and four others are related to the effects of the nuclear receptor (SR). All drug molecules with original SMILES representations are transformed into quantitative descriptor representations using the Modred descriptor calculator [26]. Under the same experimental conditions and settings, we compared our proposed BACO approach with five well-known feature selection methods, including the chi-square test (CHI) [27], Gini index (Gini) [28], minimum-redundancy-maximum-relevance (mRMR) [29], mutual information (MI) [30], and ReliefF [31], which are also widely used feature selection techniques in QSAR [10,11]. The experimental results show that BACO obviously outperforms several competitors on skewed Tox21 datasets when selecting the same number of features, indicating its effectiveness on unevenly distributed data. Additionally, the experimental results illustrate that, by using BACO, the best performance can be obtained with only a few to dozens of descriptors on most datasets.

The rest of this study is organized as follows. Section 2 provides the experimental results and presents the corresponding discussions. In Section 3, the Tox21 datasets, Modred descriptor representation, and proposed BACO method are described in detail. Section 4 summarizes the findings of the study.

## 2. Results and Discussion

This section aims to verify the effectiveness and superiority of the proposed BACO algorithm using experiments. First, the results based on the initially screened features and high-frequency features acquired by BACO were compared with show the effect of feature selection. Then, based on the same experimental settings, the BACO algorithm was compared with several benchmark feature selection algorithms, aiming to show the superiority of BACO. Next, we explored the impact of the number of selected features *K* on the BACO algorithm. Furthermore, we investigated the impact of adopting several different basic classifiers and determined the generalizability and applicability of the BACO algorithm by verifying it on three other imbalanced feature selection and classification tasks. Finally, the top 20 high-frequency descriptors on each Tox21 dataset were listed to explain why they help to effectively distinguish active/inactive molecules; we further provide several suggestions about how to use them to enhance our understanding of the relationship between drug toxicity and various molecule structure features, as well as in drug molecule design. It is specifically noted that all experimental results are shown as the mean of 10 random five-fold cross-validations, which helps to avoid providing incorrect evaluations for various methods.

### 2.1. Comparison Between Initially Screened Features and High-Frequency Features

As we know, the mechanism of multiple random divisions adopted by BACO produces many sub-optimal feature subsets. Each feature that emerged in these subsets can help distinguish active/inactive molecules to some extent, and the features that are present in the same subset can cooperate with each other well. This raises the question of whether combining all features emerging in *K* optimal subsets could yield good enough classification performance or whether selecting a few high-frequency features from these subsets could produce a better performance. Table 1 presents the classification performance based on the initially screened features and the top 20 high-frequency features acquired from BACO.

The results in Table 1 show that adopting a few high-frequency features can significantly improve the performance of the classification model as compared with using the initially screened features. Specifically, on nearly all datasets, the classification performance of BACO based on the top 20 features was improved. We believe that, although each initially screened feature comes from at least one optimal feature subset, there may exist very severe redundancy as each feature subset was acquired from one individual division. In addition, some features with weak distinguishing abilities were also put into the initially screened feature set. These two reasons explain why directly using the initially screened features cannot yield excellent performance. In our BACO, the high frequency not only represents a high distinguishing ability but also indicates the significance of the corresponding feature. Therefore, combining a few high-frequency features helps to promote the performance of a learning model. The results presented in Table 1 preliminarily verify the effectiveness of the proposed BACO algorithm.

### 2.2. Comparison Between BACO and Several Benchmark Feature Selection Approaches

Next, we compared the performance of BACO and several benchmark feature selection algorithms, which were mentioned in Section 1. Specifically, to guarantee the impartiality of the experimental comparison, all feature selection algorithms selected the top 20 features. The experimental results are presented in Table 2, Table 3, Table 4, Table 5 and Table 6, where the best results have been highlighted in bold.

The results in Table 2, Table 3, Table 4, Table 5 and Table 6 show that, for all datasets except DS7, BACO yields better results than five widely used feature selection approaches, both in terms of the F-measure and G-mean metrics. In addition, we observed that, for the MCC metric, BACO yielded the best results on nine datasets but produced slightly worse results on DS8 than CHI and on DS12 than mRMR. As for the AUC and PR-AUC metrics, BACO yielded the best results on 11 and 10 datasets, respectively, with results that were significantly superior to those of the five other feature selection methods. It is particularly noteworthy that both the AUC and PR-AUC metrics did not participate in the optimization procedure of BACO. Their improvement indicates the credibility of the selected features using this method, as they can truly improve classification performance in the context of imbalanced data. The results above also further indicate the effectiveness and superiority of the proposed BACO algorithm. It is not difficult to understand these results, as all other feature selection algorithms fail to consider the associations among those selected top *K* features, as well as the influence of imbalanced data distribution. For feature selection, a worse result is often produced by combining those features with the most distinguishing abilities rather than integrating some important features to support each other. Additionally, an extremely high-class imbalance distribution may lower the accuracy of most traditional feature selection methods. That explains why our proposed BACO approach exhibits obviously improved performance in comparison to several benchmark feature selection methods. Moreover, we note that except for mRMR, the other compared methods fail to remove redundant features, which explains why mRMR behaves better than the four other methods.

**Figure 1:**
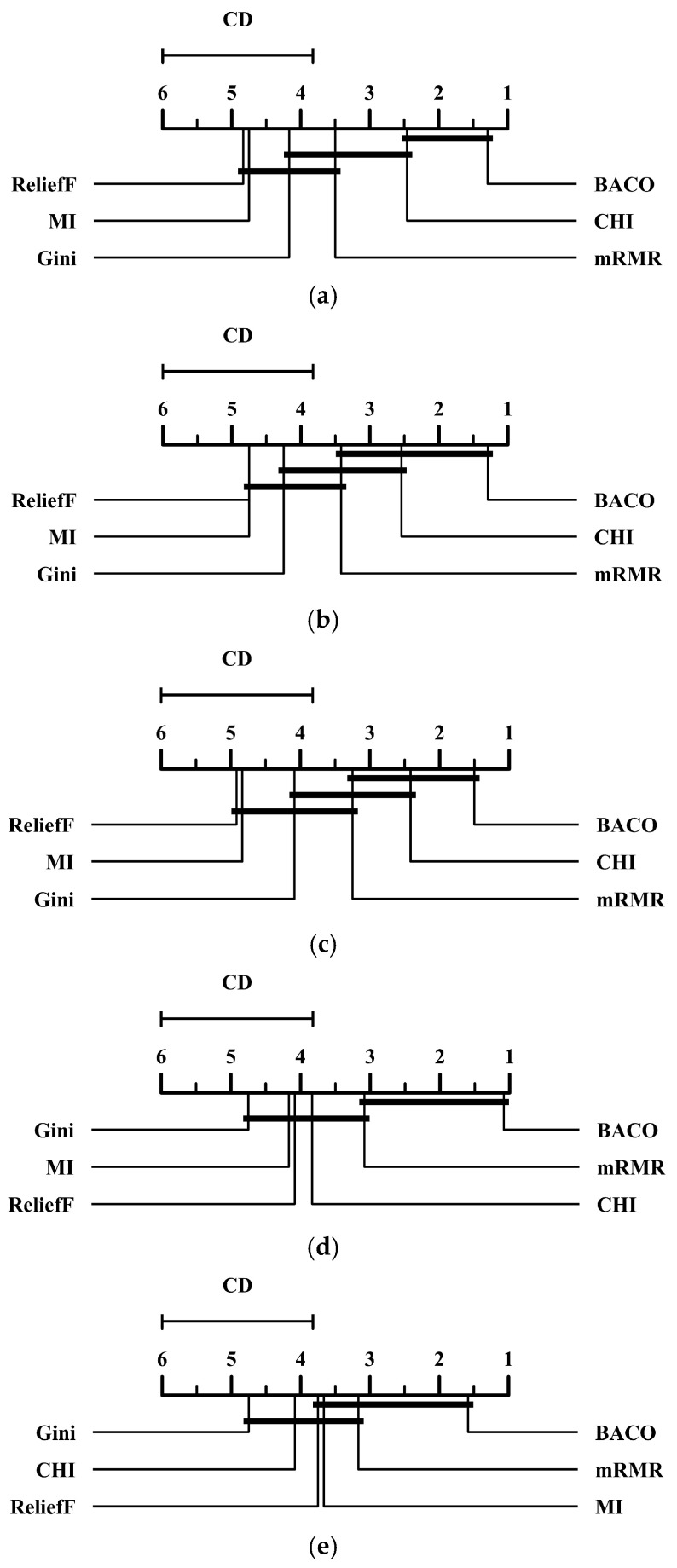
CD diagrams of several feature selection methods at a standard level of significance α = 0.05 in terms of five performance metrics. (**a**) CD diagram of the F-measure metric. (**b**) CD diagram of the G-mean metric. (**c**) CD diagram of the MCC metric. (**d**) CD diagram of the AUC metric. (**e**) CD diagram of the PR-AUC metric.

To confirm the superiority of the proposed BACO feature selection approach, we also conducted the Nemenyi test [32,33] for the results in Table 2, Table 3, Table 4, Table 5 and Table 6. Figure 1 presents the critical difference (CD) diagrams of several feature selection methods at a standard level of significance α = 0.05 in terms of five different performance metrics. Specifically, if the difference between the average rankings belonging to two different methods is lower than a CD unit, then they would be regarded as having no significant difference in statistics. From Figure 1, we observe that BACO significantly outperforms several other methods, except for CHI and mRMR, in terms of both the G-mean and MCC metrics. In both the F-measure and AUC metrics, BACO shows no significant difference with only one method, CHI and mRMR, respectively. Meanwhile, for the PR-AUC metric, BACO only significantly outperforms two other feature selection methods, namely, Gini and CHI. To summarize, although the superiority of BACO is not significant enough in comparison with a few methods, it can still be regarded as the best option among these compared approaches as it yields the lowest average ranking on each performance metric. 

### 2.3. Impact of the Parameter K on the Performance of BACO

In the second layer of BACO, the top *K* high-frequency features must be extracted to train the final classification model. Then, we have to address the question of how to provide an appropriate setting for *K*. Next, we tested the impact of the parameter K on the performance of BACO. Here, we varied K in the range of {5, 10, 20, 30, 50, 100, 200, 300}, and the results are presented in Table 7 and Tables S1 and S2, where the best results for each dataset are highlighted in bold.

The results in these tables show that, for most datasets, selecting a few or dozens of features with BACO is guaranteed to yield excellent classification performance. Specifically, on the DS4 dataset, training the classification model on the top five features yields the best performance. The results illustrate that only a few descriptors are associated with whether a drug molecule exhibits toxicity. If designating an extremely large *K* value in BACO, some weak, relevant, and redundant descriptors will destroy the classification model and further lower its quality. By analyzing the experimental results, we recommend designating K with a value no larger than 100 in practical applications.

### 2.4. Impact of Adopting Different Basic Classifiers in BACO

Next, we deemed it necessary to investigate the impact of basic classifiers in BACO [34]. In addition to SVM, we compared the performance of four other representative classifiers, including a classification and regression tree (CART) [35], logistic regression (LR) [36], random forest (RF) [37], and XGBoost [38]. The compared results are presented in Table 8 and Tables S3 and S4; for each dataset, the best result in terms of each metric is highlighted in bold.

The results in Table 8 and Tables S3 and S4 show that among several compared classifiers, SVM, RF, and XGBoost yield the best results in terms of all combinations of performance metrics and datasets. This indicates that, when integrating one of these three classifiers into the optimization procedure of BACO, it tends to achieve better performance than when integrating two others. As we know, SVM, RF, and XGBoost are generally more robust and stable in the context of imbalanced data distribution than the two others, and thus, we cannot say that adopting CART and LR yields worse feature selection results, as the difference in classification performance may be driven by the difference among classifiers. When applying BACO in a practical feature selection task, we suggest selecting the appropriate classifier with regard to the requirements of classification performance and learning efficiency.

### 2.5. Detecting the Generalizability and Applicability of BACO

To verify the generalizability and applicability of BACO, we tested it on three other datasets, including two ovarian mass spectrometry datasets (Ovarian I and Ovarian II) [39] and a colon cancer microarray gene expression dataset (Colon) [40]. Specifically, both the Ovarian I and Ovarian II datasets contain 116 mass spectrometry instances derived from the serum of women. The task of Ovarian I is to distinguish 16 benign samples from 100 ovarian cancer examples, while Ovarian II is used to distinguish the same 16 benign samples from 100 unaffected examples; i.e., in these two datasets, the minority class represents 13.8% of all instances. Each sample is represented by 15,154 features. The Colon dataset contains 62 samples collected from colon cancer patients. Among them, 40 tumor biopsies are from tumors, and 22 normal biopsies are from healthy parts of the colons of the same patients, i.e., the minority class represents 35.5% of all instances. Each instance is represented by 2000 features. Considering the high-dimensional and small sample characteristics of these datasets, we manually tuned several default parameters to adapt them, including tuning the iteration rounds *R* from 10 to 100 to guarantee sufficient optimization and tuning the initial pheromone concentration in pathway 1 *ph_ini1_* to rapidly focus on a few highly relevant features with classification.

Similarly to the experiments conducted on the Tox21 datasets, we selected the top 20 features using various feature selection methods and then compared their performance in terms of the F-measure, G-mean, MCC, AUC, and PR-AUC. The results are presented in Table 9.

The results in Table 9 show that, when applied to three other high-dimensional and small-sample datasets, BACO can still yield significantly better performance than several other feature selection methods. High dimensionality generally means that some pseudo-significant features are easily extracted, but this is not true of the strongly relevant ones; meanwhile, a small sample tends to provide unstable feature selection results. In BACO, although these two data characteristics may cause a very large distribution drift between two random divisions, further influencing the quality and stability of feature selection, these issues can be effectively addressed by simultaneously decreasing the initial pheromone concentration in pathway 1 and increasing the number of iterations and divisions. According to the experimental results above, we can conclude that BACO possesses strong generalizability and applicability, guaranteeing the quality of feature selection in various scenarios.

### 2.6. Discussion and Further Suggestions

Finally, to further understand the association between molecule toxicity and molecule structure, the information about the top 20 high-frequency descriptors acquired by BACO on each dataset is listed. The information helps us to determine which structural properties and their corresponding physicochemical or biological properties directly induce toxicity, further helping us to develop more accurate prediction models and to design drug molecules without toxicity. Specifically, we list the information of the top 20 high-frequency descriptors, except DS7, on all of the datasets in Table 10 and Tables S5–S14.

Specifically, we note that some descriptors frequently emerge in high-frequency lists of various molecular pathway endpoints, including nG12FARing, nG12Fring, n5ARing, nBridgehead, SlogP_VSA6, and SRW05.

Among these descriptors, both nG12FARing and nG12Fring denote a large ring size, which has also been found to be closely associated with molecule toxicity in some previous research [41,42]. A large ring size helps to significantly increase molecule size and volume, which can hinder efficient excretion via the renal or hepatic pathways, meaning that they are retained in the body and contribute to prolonged toxic effects. In addition, a large ring structure tends to increase the hydrophobic surface area of a molecule, further promoting non-covalent interactions with the hydrophobic regions of proteins or nucleic acids. These interactions may disrupt the structural integrity of biomolecules, leading to misfolding, aggregation, or loss of function.

As for n5ARing, it quantifies the number of aromatic rings with five members (e.g., furan, thiophene, pyrrole, or cyclopentadienyl anion) in a molecule. It has been found that n5ARing can increase the potential for reactive metabolite formation, further leading to cellular damage; it enhances binding affinity to biological targets, potentially disrupting normal cellular functions, and influences molecular polarity and solubility, further affecting the distribution and accumulation of the compound in biological systems [43].

nBridgehead denotes the number of bridgehead atoms in a molecule. The bridgehead atoms can increase the rigidity of a molecule to limit its flexibility and conformational changes while enhancing the metabolic stability of a molecule and its hydrophobicity, and they may also introduce additional steric hindrance [44]. It has been observed that the nBridgehead property is helpful for distinguishing toxic from non-toxic compounds based on several potential reasons: it enhances the metabolic stability and bioaccumulation of the molecule, further prolonging its activity in the body; it may introduce structural rigidity and steric hindrance, further interfering with normal biological processes; and it can influence the hydrophobicity and electronic properties of the molecule, further leading to adverse interactions with biological targets [45].

SlogP_VSA6 describes the van der Waals surface area of specific hydrophobic regions on the molecular surface; thus, it can capture the key features of molecular hydrophobicity and surface polarity, which directly influence the molecule’s biodistribution, metabolism, and interactions with biological targets, thereby determining its toxic potential [46,47].

SRW05 describes the topological features of a molecule with a self-returning walk length of five, reflecting structural information in three-dimensional space. These features indirectly influence the shape, flexibility, steric hindrance, and metabolic pathways of the molecule, thereby determining its toxic potential [48,49].

Readers are encouraged to use the structural features and physicochemical and/or biological properties reflected by the significant descriptors acquired from BACO to research and understand the relationship between molecule structure and compound toxicity in their specific experiments.

In addition, the significant descriptors selected by BACO can be used to design and modify chemical structures, with the aim of reducing molecule toxicity and accelerating drug design. Taking the partial significance descriptors discussed above as an example, we suggest adjusting the large ring counts to decrease the molecule’s hydrophobic surface area and optimize ring connectivity in order to control the number of five-membered aromatic rings to decrease the potential for reactive metabolite formation and reduce the likelihood of generating toxic intermediates. This will reduce the number of bridgehead atoms in order to decrease molecular rigidity and optimize the chemical environment, optimizing hydrophobicity to balance hydrophilicity/lipophilicity and modify the topological structure of the molecule or introduce flexible groups to minimize interactions with off-target molecules. Further molecular modification for toxicity control and practical drug design requires QSAR analysis based on molecular toxicity information. In addition, the descriptors acquired from BACO can only help to accelerate drug design: the actual toxicity of a designed chemical must still be measured using in vitro toxicity testing.

## 3. Materials and Methods

This section first describes the details of Tox21 datasets used in the experiments and then explains how to use the Modred descriptor calculator to represent drug molecules in datasets. Next, the BACO feature selection algorithm is introduced in detail.

### 3.1. Datasets and Their Representations

#### 3.1.1. Tox21 Datasets

In this study, twelve Tox21 datasets are used. These datasets come from the Tox21 Data Challenge, which is an open-access resource that aims to help drug developers understand the chemical toxicology that can disrupt biological pathways and further induce toxic effects. The toxic effects included in the twelve Tox21 datasets refer to the stress response (SR) and the effects of the nuclear receptor (NR). Specifically, among the twelve datasets, eight refer to NR, and four others relate to SR. Toxic effects activated by SR pathways tend to damage the liver and even cause cancer, while the toxic effects activated by NR pathways can disrupt the functions of the endocrine system.

The details of the twelve Tox21 datasets [25] are presented in Table 11, in which # molecules denotes the number of drug molecules, and # inactive and # active, respectively, represent the number of inactive and active drug molecules in the corresponding dataset. In addition, we provide statistics about the proportion of active molecules among all molecules and give molecular pathway endpoint descriptions in each dataset. It is not difficult to observe that all of the datasets are imbalanced to some extent, as the statistic about the ratio of active molecules ranges between 2.6% and 15.8%. This explains why class imbalance issues must be considered in tasks predicting the toxicity of drug molecules.

#### 3.1.2. Modred Descriptor Calculator

In this study, each drug molecule is represented as a vector that is sequentially constituted by 1610 two-dimensional descriptors. This task is accomplished using the Modred descriptor calculation software (https://github.com/mordred-descriptor, accessed on 6 October 2024) [26], which is freely available, fast, and able to calculate descriptors for large molecules. Using the Modred descriptor calculator, all drug molecules with original SMILES representations can be transformed into quantitative descriptor representations. The specific descriptor information is given in Table 12.

### 3.2. Binary Ant Colony Optimization (BACO) Feature (Descriptor) Selection Algorithm

#### 3.2.1. Optimal Feature Group Search Using BACO

The ant colony optimization (ACO) algorithm proposed by Dorigo et al. [17] is a well-known swarm optimization algorithm that has been widely used to deal with various real-world optimization issues, especially discrete ones. Specifically, ACO simulates the foraging behavior of ant colonies. During foraging, ants communicate with each other by releasing pheromones into the air since they tend to assemble in locations with high pheromone concentrations. In addition, the pheromone evaporates over time. Based on these basic conditions, ant colonies present intelligent behavior that is not possessed by a single ant.

**Figure 2:**
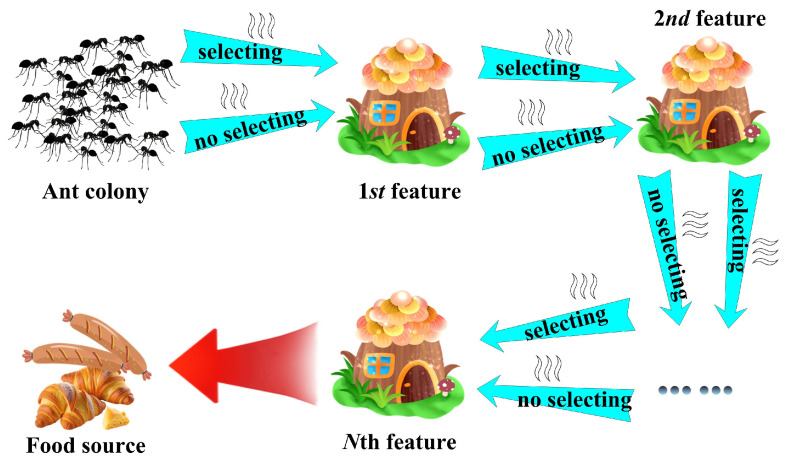
Mechanism procedure of the BACO algorithm.

In this study, we design a binary ant colony optimization (BACO) algorithm to conduct feature selection tasks. The mechanism procedure of BACO is described in Figure 2. It can be observed that, between the nest and the food source, there are *N* sequentially arranged sites, with each one corresponding to a feature. From one feature to the next, there are two alternative pathways: one denotes that the next feature should be extracted into the feature group, and the other one denotes the next feature should be abandoned. When an ant travels from one feature to the next feature, the probability of selecting the pathway *j* (*j* = 1 or 2) can be calculated as follows:

(1) $$P_{ij}=\frac{\tau_{ij}}{\sum_{j=1}^{2}\tau_{ij}}$$

where *i* denotes the *i*th feature, while $\tau_{ij}$ and $P_{ij}$ represent the pheromone concentration of the *j*th pathway and the probability of selecting the *j*th pathway of the *i*th feature by an ant, respectively. Specifically, to avoid selecting an excessive number of features into the subset, a higher initial pheromone concentration should be pre-assigned for pathway 2 than for pathway 1. Next, after all *S* ants have finished their journeys, *S* feature subsets can be acquired according to their choices about the pathways. Based on these *S* feature subsets, *S* SVM classifiers are trained on the training sets, and then their quality can be further evaluated on the validation sets using the following fitness function:

(2) fitness =α×F-measure+β×G-mean+γ×MCCst. α+β+γ=1 

where *α*, *β*, and *γ* are weights for three different performance metrics, i.e., the F-measure, G-mean, and MCC, and their summation is 1. Specifically, the calculation of these three performance metrics relies on the fusion matrix illustrated in Table 13. Here, *TP*, *TN*, *FP*, and *FN* are statistics used to record the number of accurately and falsely classified instances belonging to the positive class and negative class, respectively. Furthermore, we can use them to calculate several metrics as follows:

(3) $$Precision(Pre)=\frac{TP}{TP+FP}$$

(4) $$Sensitivity(Sen)=\frac{TP}{TP+FN}$$

(5) $$Specificity\left(Spe\right)=\frac{TN}{TN+FP}$$

Next, the F-measure, G-mean, and MCC metrics can be calculated as follows:

(6) $$F-measure=\frac{2\times Pre\times Sen}{Pre+Sen}$$

(7) $$G-mean=\sqrt{Sen\times Spe}$$

(8) $$MCC=\frac{TP\times TN-FP\times FN}{\sqrt{\left(TP+FN\right)\times \left(TN+FN\right)\times \left(TP+FP\right)+\left(TN+FP\right)}}$$

It is clear that all three of these performance metrics evaluate the quality of a learning model in the context of imbalanced data. Thus, the fitness function can reflect the value of the features that help to improve the quality of a learning model when it is applied to skewed data. Aside from these three metrics, both the area under ROC curves (AUC) [50] and precision–recall AUC (PR–AUC) [51] have also been widely used to evaluate the quality of a learning algorithm in the context of imbalanced data. In this study, we did not use them in our fitness function for optimization, but they are used to reflect the real quality of the selected features in our experiments.

Furthermore, based on the fitness evaluation, the pheromone concentration of each pathway is updated using the following function:

(9) $$\tau_{ij}\left(t+1\right)=(1-\rho )\times \tau_{ij}\left(t\right)+\Delta \tau_{ij}$$

where ρ∈(0,1) denotes the evaporation factor, t corresponds to the iteration number of BACO, and $\Delta \tau_{ij}$ represents the pheromone concentration increment on the corresponding pathway. Here, we only added the pheromone concentration corresponding to the pathways emerging in the best 10% of ants. Specifically, we stored the best pathways in a set *E*. Then, $\Delta \tau_{ij}$ can be calculated as follows:

(10) $$\Delta \tau_{ij}=\left\{\begin{array}{c} \frac{1}{0.1\times S}\times fitness,{\mathrm{pathway}}_{ij}\in E\\ 0,\;{\mathrm{pathway}}_{ij}\notin E \end{array}\right.$$

According to Equations (9) and (10), after an iteration is finished, the poor pathways are weakened in terms of pheromone concentration by the introduction of the evaporation factor ρ, while the good pathways are intensified to promote the probability of selecting them in the next iteration. To avoid acquiring an overfitting result, the lower bound ${ph}_{min}$ and upper bound ${ph}_{max}$ of pheromone concentration in each pathway are also pre-designated.

BACO repeats the above optimization procedure until the pre-defined number of iterations is satisfied. Finally, the optimal feature group that corresponds to the highest fitness throughout the optimization procedure is generated.

#### 3.2.2. Filter Feature Selection Based on Frequency Statistics Acquired by BACO

When using a fixed training set and validation set, the data distribution may deviate from the real one, and limited instances may influence the stability of feature selection; this leads BACO to generate an overfitting feature subset. To address this issue, we adopt a strategy of multiple divisions to evaluate the significance of each feature. Specifically, a five-fold cross-validation approach is adopted to divide the training set and testing set, and then the training set is randomly divided into a training subset, and a validation subset based on the ‘8–2 rule’, i.e., 80% of instances are assigned into the training subset, and the remaining 20% of instances are placed into the validation subset. BACO runs on each group of divisions and outputs the optimal feature group. Next, a filter feature selection procedure based on the statistics of the emerging frequency of each feature in multiple sub-optimal feature groups is conducted to extract the *K* most significant features. Finally, we only use these *K* features on the original training set to train the SVM classifier and verify its performance on the testing set. A similar Bootstrap approach [52], using a multiple random division strategy, is expected to produce more stable feature selection results on even smaller sample datasets.

**Figure 3:**
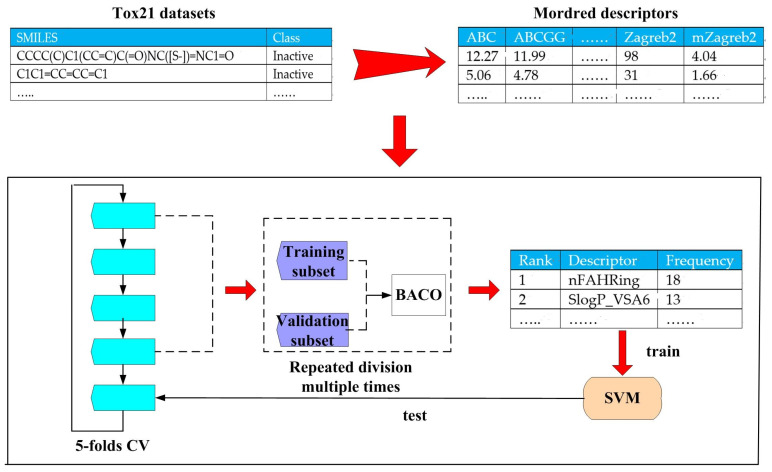
Filter feature selection and toxicity prediction procedure based on BACO.

Taking the toxicity prediction task as the object, our filter feature selection and classification procedure is fully described in Figure 3. Specifically, BACO represents a filter feature selection method that relies on the statistical results of multiple wrapper feature selection procedures.

#### 3.2.3. Default Parameter Settings and Time Complexity Analysis

Table 14 presents the default parameter settings used in this study. Specifically, the iteration times of BACO *R* are empirically designated as 10 for the two following reasons: (1) in most cases, this setting is guaranteed to converge on an optimal solution, and (2) the optimization process of BACO is relatively time-consuming. As for the number of random divisions *M*, we provided a medium setting for it on the basis that it could simultaneously highlight the significant features and save time.

Next, the time complexity of the BACO algorithm is analyzed in detail. In each minimal iteration, generating ants requires consuming *O*(*NS*), training SVMs for these ants costs *O*(*h^2^NS*)~*O*(*h^3^NS*), and acquiring the prediction performance consumes *O*(*h^2^NS*) time, where *h* denotes the number of instances and *N* denotes the number of features. In total, there are *R* iterations for each ant and *M* iterations. Therefore, the time complexity of BACO based on SVM constructing the RBF kernel function is between *O*(*h^2^NSRM*) and *O*(*h^3^NSRM*). Thus, it is extremely time-consuming in comparison with filter-based feature selection methods. In some other practical applications, we suggest users tune the number of ants *S*, the iteration times of BACO *R*, and the number of random divisions *M* according to the data’s internal characteristics, further reducing its running time. In addition, the user should also adopt classifiers with low time complexity when they are working with large datasets; alternatively, they can lower the initial pheromone concentration in pathway 1, which helps to significantly lower the feature dimensions of the estimated subset corresponding to each ant when the dataset is extremely high-dimensional. This will reduce the running time of BACO. All in all, BACO is a time-consuming feature selection method; however, in our opinion, it can be used for static feature selection and prediction tasks, as the accuracy of the algorithm is always more important than its real-time requirements.

#### 3.2.4. Applicability and Limitations of BACO

In theory, BACO is appropriate for use in various feature selection scenarios, even when feature distribution is manifold, as it evaluates the significance of features in an indirect fashion based on the performance feedback from the classifier. It can be used to adapt various feature selection targets by modifying the fitness function, e.g., when the data distribution is balanced, the fitness function can be replaced by the accuracy metric. Alternatively, when it encounters a regression task, the fitness function can be modified as MSE or MAE losses. Therefore, BACO is both a robust and universal feature selection algorithm.

However, it may encounter some limitations in specific application scenarios. First, it is relatively sensitive to the size of the training set; for such datasets, it tends to output a local optimal result, although the issue can be alleviated by increasing the number of internal divisions. In addition, it is difficult to apply BACO to large-scale high-dimensional data; in these situations, it is not easy for BACO to find the optimal tradeoff between feature selection quality and running time. However, on datasets such as Tox21 that only contain thousands of instances and hundreds of features, BACO can easily produce high-quality feature selection within a limited time.

## 4. Conclusions

In this study, we proposed a novel feature selection method, BACO, which is based on the idea of ant colony optimization for predicting the toxicity of drug molecules. Specifically, BACO simultaneously focuses on the challenges presented by the skewed data distribution of drug activity data and the issue of dimensionality. To adapt to the skewed data distribution, BACO adopts a combination of three performance metrics, which are specifically designed to evaluate the quality of learning models on imbalanced data as the fitness function. To guarantee the quality and stability of feature selection, we designed a feature frequency ranking mechanism based on multiple random divisions, which can effectively reduce the negative effects caused by local optimization searches. The experimental results for the 12 datasets in the Tox21 challenge illustrate that the proposed BACO method significantly outperforms traditional feature selection approaches when selecting only a few or dozens of descriptors, indicating its effectiveness and superiority. It can be regarded as an effective tool in various molecule activity prediction tasks.

## Figures and Tables

**Table 1:** Performance comparison between initially screened features (covering all features emerging in *K* optimal feature subsets) and high-frequency features (top 20).

Dataset	Initially Screened Features						High-Frequency Features					
	# Descriptors	F-Measure	G-Mean	MCC	AUC	PR-AUC	# Descriptors	F-Measure	G-Mean	MCC	AUC	PR-AUC
DS1	672	0.5519	0.6467	0.5727	0.7128	0.0897	20	0.6029	0.6866	0.6170	0.7657	0.1616
DS2	669	0.5732	0.6790	0.5819	0.7931	0.0732	20	0.6168	0.7286	0.6142	0.8336	0.1033
DS3	672	0.0898	0.2173	0.1809	0.6659	0.1758	20	0.2334	0.3779	0.2568	0.7529	0.2595
DS4	671	0.0000	0.0000	0.0000	0.6178	0.0597	20	0.0570	0.1509	0.1365	0.6856	0.1191
DS5	670	0.0437	0.1484	0.1389	0.5993	0.1571	20	0.1997	0.3367	0.2722	0.7198	0.2948
DS6	670	0.0000	0.0000	0.0000	0.6037	0.0614	20	0.1465	0.2801	0.2488	0.6854	0.1732
DS7	670	0.0000	0.0000	0.0000	0.6227	0.0391	20	0.0000	0.0000	0.0000	0.6496	0.0547
DS8	629	0.0000	0.0000	0.0000	0.5949	0.1732	20	0.0884	0.2174	0.1345	0.8159	0.2193
DS9	671	0.0000	0.0000	0.0000	0.5558	0.0588	20	0.0236	0.0848	0.0833	0.6434	0.1490
DS10	629	0.0000	0.0000	0.0000	0.6337	0.0742	20	0.0311	0.0970	0.0947	0.7225	0.1229
DS11	672	0.1302	0.2579	0.2018	0.7907	0.2335	20	0.2722	0.4110	0.2816	0.8471	0.3845
DS12	670	0.0000	0.0000	0.0000	0.5892	0.0698	20	0.0620	0.1778	0.1142	0.6778	0.1871

# Descriptors denote the number of features (descriptors).

**Table 2:** Classification performance of BACO and several benchmark feature selection methods in terms of the F-measure metric.

Dataset	CHI	Gini	mRMR	MI	ReliefF	BACO
DS1	0.5959	0.5545	0.5366	0.5570	0.4379	**0.6029**
DS2	0.6060	0.5591	0.5543	0.5774	0.5586	**0.6168**
DS3	0.1717	0.0776	0.1738	0.0028	0.0287	**0.2334**
DS4	0.0394	0.0131	0.0384	0.0000	0.0000	**0.0570**
DS5	0.1014	0.0000	0.0639	0.0000	0.0000	**0.1997**
DS6	0.1247	0.0395	0.1148	0.0000	0.0000	**0.1465**
DS7	0.0000	0.0000	0.0000	0.0000	0.0000	0.0000
DS8	0.0756	0.0000	0.0000	0.0000	0.0068	**0.0884**
DS9	**0.0236**	0.0000	**0.0236**	0.0000	0.0000	**0.0236**
DS10	0.0098	0.0000	0.0000	0.0000	0.0000	**0.0311**
DS11	0.1877	0.2264	0.2115	0.0000	0.0421	**0.2722**
DS12	0.0346	0.0000	0.0541	0.0000	0.0000	**0.0620**

**Table 3:** Classification performance of BACO and several benchmark feature selection methods in terms of the G-mean metric.

Dataset	CHI	Gini	mRMR	MI	ReliefF	BACO
DS1	0.6827	0.6434	0.6375	0.6478	0.5273	**0.6866**
DS2	0.7107	0.6753	0.6688	0.6858	0.6804	**0.7286**
DS3	0.3133	0.2003	0.3144	0.0167	0.1187	**0.3779**
DS4	0.1085	0.0517	0.1221	0.0000	0.0000	**0.1509**
DS5	0.2306	0.0000	0.1803	0.0000	0.0000	**0.3367**
DS6	0.2546	0.0896	0.2447	0.0000	0.0000	**0.2801**
DS7	0.0000	0.0000	0.0000	0.0000	0.0000	0.0000
DS8	0.1978	0.0000	0.0000	0.0000	0.0451	**0.2174**
DS9	**0.0848**	0.0000	**0.0848**	0.0000	0.0000	**0.0848**
DS10	0.0446	0.0000	0.0000	0.0000	0.0000	**0.0970**
DS11	0.3274	0.3652	0.3469	0.0000	0.1370	**0.4110**
DS12	0.1032	0.0000	0.1636	0.0000	0.0000	**0.1778**

**Table 4:** Classification performance of BACO and several benchmark feature selection methods in terms of the MCC metric.

Dataset	CHI	Gini	mRMR	MI	ReliefF	BACO
DS1	0.6106	0.5803	0.5541	0.5790	0.4451	**0.6170**
DS2	0.6090	0.5653	0.5630	0.5849	0.5603	**0.6142**
DS3	0.2166	0.1606	0.2254	0.0157	0.1056	**0.2568**
DS4	0.1057	0.0503	0.1189	0.0000	0.0000	**0.1365**
DS5	0.2064	0.0000	0.1653	0.0000	0.0000	**0.2722**
DS6	**0.2488**	0.0875	0.2391	0.0000	0.0000	**0.2488**
DS7	0.0000	0.0000	0.0000	0.0000	0.0000	0.0000
DS8	**0.1356**	0.0000	0.0000	0.0000	0.0389	0.1345
DS9	**0.0833**	0.0000	**0.0833**	0.0000	0.0000	**0.0833**
DS10	0.0418	0.0000	0.0000	0.0000	0.0000	**0.0947**
DS11	0.2372	0.2694	0.2512	0.0000	0.0901	**0.2816**
DS12	0.0875	0.0000	**0.1408**	0.0000	0.0000	0.1142

**Table 5:** Classification performance of BACO and several benchmark feature selection methods in terms of the AUC metric.

Dataset	CHI	Gini	mRMR	MI	ReliefF	BACO
DS1	0.7436	0.7099	0.7528	0.7244	0.7497	**0.7657**
DS2	0.7887	0.7792	**0.8429**	0.8007	0.7861	0.8336
DS3	0.6730	0.6701	0.6733	0.6728	0.7125	**0.7529**
DS4	0.6766	0.6429	0.6407	0.6541	0.6250	**0.6856**
DS5	0.7046	0.6288	0.6773	0.6332	0.6590	**0.7198**
DS6	0.6468	0.6577	0.6680	0.6145	0.6399	**0.6854**
DS7	0.6081	0.6346	0.6229	0.6458	0.6335	**0.6496**
DS8	0.7032	0.6758	0.7327	0.7565	0.7021	**0.8159**
DS9	0.6218	0.5421	0.6036	0.5976	0.5808	**0.6434**
DS10	0.5979	0.6544	0.6770	0.6231	0.6080	**0.7225**
DS11	0.7499	0.8006	0.8138	0.7999	0.8302	**0.8471**
DS12	0.6656	0.6247	0.6242	0.6108	0.6334	**0.6778**

**Table 6:** Classification performance of BACO and several benchmark feature selection methods in terms of the PR-AUC metric.

Dataset	CHI	Gini	mRMR	MI	ReliefF	BACO
DS1	0.1412	0.1009	0.1458	0.0950	0.1132	**0.1616**
DS2	0.1330	0.1217	**0.1448**	0.0979	0.0856	0.1033
DS3	0.1810	0.2009	0.2032	0.2406	0.2129	**0.2595**
DS4	0.0228	0.0240	0.0775	0.0957	0.0656	**0.1191**
DS5	0.2369	0.1952	0.2244	0.2667	0.2324	**0.2948**
DS6	0.0838	0.0992	0.1348	0.1252	0.1387	**0.1732**
DS7	0.0556	**0.0622**	0.0617	0.0438	0.0610	0.0547
DS8	0.1991	0.1846	0.2033	0.1996	0.2148	**0.2193**
DS9	0.1336	0.1028	0.0793	0.0881	0.0742	**0.1490**
DS10	0.0889	0.0795	0.1034	0.0929	0.1036	**0.1229**
DS11	0.2827	0.2541	0.3136	0.3376	0.3051	**0.3845**
DS12	0.1141	0.0782	0.0914	0.1081	0.0905	**0.1871**

**Table 7:** Classification performance of BACO on the DS1~DS4 datasets with different *K* settings.

Number of Selected Descriptors *K*	F-Measure	G-Mean	MCC	AUC	PR-AUC
DS1					
5	0.5737	0.6699	0.5867	0.6759	0.0917
10	0.5968	0.6861	0.6082	0.7421	0.1356
20	0.6029	0.6866	0.6170	0.7657	0.1616
30	0.6041	0.6886	0.6177	0.7698	0.1681
50	0.6087	0.6941	0.6214	0.7736	0.1744
100	0.6085	0.6941	0.6213	**0.8105**	0.1752
200	0.6110	0.6962	0.6235	0.8032	**0.1810**
300	**0.6153**	**0.6987**	**0.6278**	0.7764	0.1732
DS2					
5	0.5722	0.7189	0.5642	0.8059	0.0692
10	0.6164	**0.7295**	**0.6162**	**0.8357**	**0.1421**
20	**0.6168**	0.7286	0.6142	0.8336	0.1033
30	0.6090	0.7204	0.6079	0.8311	0.1058
50	0.6073	0.7203	0.6060	0.8230	0.1011
100	0.6136	0.7206	0.6138	0.8167	0.0872
200	0.6156	0.7206	0.6131	0.8123	0.0903
300	0.6139	0.7205	0.6141	0.8056	0.0887
DS3					
5	0.1568	0.2985	0.2053	0.6577	0.1830
10	0.2123	0.3557	0.2395	0.7121	0.2093
20	0.2334	0.3779	0.2568	0.7529	**0.2595**
30	0.2612	**0.4030**	0.2817	**0.7636**	0.2546
50	**0.2651**	0.4027	**0.2997**	0.7519	0.2319
100	0.2200	0.3603	0.2602	0.7253	0.2172
200	0.1990	0.3385	0.2564	0.7034	0.2005
300	0.1480	0.2863	0.2134	0.6908	0.1891
DS4					
5	**0.0958**	**0.2190**	**0.2082**	**0.7236**	**0.1459**
10	0.0843	0.2046	0.1995	0.6959	0.1392
20	0.0570	0.1509	0.1365	0.6856	0.1191
30	0.0508	0.1434	0.1322	0.6758	0.1201
50	0.0515	0.1597	0.1470	0.6813	0.1096
100	0.0443	0.1325	0.1290	0.6467	0.0989
200	0.0324	0.1140	0.1110	0.6501	0.0807
300	0.0253	0.0870	0.0847	0.6288	0.0753

**Table 8:** Classification performance of BACO on the DS1~DS4 datasets with different basic classifiers.

Classifier	F-Measure	G-Mean	MCC	AUC	PR-AUC
DS1					
SVM	0.6029	0.6866	**0.6170**	**0.7657**	0.1616
CART	0.5746	0.6517	0.5842	0.7126	0.1110
LR	0.5978	0.6429	0.6011	0.6959	0.0979
RF	0.5898	0.6917	0.6154	0.7452	**0.1842**
XGBoost	**0.6232**	**0.7135**	0.6127	0.7591	0.1721
DS2					
SVM	**0.6168**	**0.7286**	**0.6142**	0.8336	**0.1033**
CART	0.5820	0.6899	0.5776	0.7984	0.0811
LR	0.5491	0.6372	0.5531	0.7521	0.0623
RF	0.6029	0.7141	0.5982	0.8419	0.0976
XGBoost	0.6171	0.7188	0.6075	**0.8501**	0.0928
DS3					
SVM	0.2334	0.3779	0.2568	**0.7529**	0.2595
CART	0.2258	0.3556	0.2426	0.6887	0.1984
LR	0.2096	0.3432	0.2581	0.6931	0.1672
RF	**0.2617**	**0.4165**	0.2607	0.7425	0.2571
XGBoost	0.2528	0.3974	**0.2753**	0.7377	**0.2692**
DS4					
SVM	**0.0570**	0.1509	0.1365	0.6856	0.1191
CART	0.0296	0.1279	0.1128	0.6572	0.0992
LR	0.0479	**0.1601**	0.1306	0.6773	0.1143
RF	0.0511	0.1548	**0.1427**	0.6654	**0.1242**
XGBoost	0.0537	0.1532	0.1358	**0.7175**	0.1103

**Table 9:** Classification performance comparison of BACO and several benchmark feature selection methods on three other datasets, in which the best results have been highlighted in bold.

Metric	CHI	Gini	mRMR	MI	ReliefF	BACO
Ovarian I						
F-measure	0.5795	0.5218	0.5944	0.5650	0.5717	**0.7201**
G-mean	0.6773	0.6379	0.7286	0.6582	0.6470	**0.8197**
MCC	0.5169	0.4822	0.4935	0.4278	0.4040	**0.6337**
AUC	0.9121	0.9038	0.9455	0.9256	0.9319	**0.9720**
PR-AUC	0.4752	0.3230	0.4438	0.4196	0.5133	**0.5872**
Ovarian II						
F-measure	0.4916	0.4783	0.4928	0.5048	0.4732	**0.6131**
G-mean	0.5742	0.5549	0.6276	0.5478	0.5947	**0.7526**
MCC	0.4554	0.4999	0.4783	0.4362	0.4881	**0.5206**
AUC	0.9570	0.9225	**0.9598**	0.9439	0.9530	0.9497
PR-AUC	0.3657	0.3571	0.3762	0.3545	0.4033	**0.4210**
Colon						
F-measure	0.7146	0.6964	0.7652	0.7277	0.7532	**0.8064**
G-mean	0.7759	0.7448	0.8118	0.7529	0.8216	**0.8688**
MCC	0.7072	0.7106	0.7529	0.6988	0.7391	**0.8179**
AUC	0.8456	0.8619	0.9098	0.8373	0.8752	**0.9137**
PR-AUC	0.6912	0.6440	0.7829	0.6827	0.7913	**0.8442**

**Table 10:** List of information about the top 20 high-frequency descriptors acquired by BACO on the DS1 dataset.

Descriptor Name	Frequency	Descriptor Definition
nG12FARing	18	Twelve-or-greater-membered aliphatic fused ring count
nG12FRing	12	Twelve-or-greater-membered fused ring count
n6ARing	11	Six-membered aliphatic ring count
nHRing	9	Hetero ring count
n5ARing	9	Five-membered aromatic ring count
SMR_VSA4	8	MOE MR VSA Descriptor 4 (2.24 ≤ x < 2.45)
nAHRing	8	Aliphatic hetero ring count
nFHRing	8	Fused hetero ring count
SRW07	7	Walk count (leg-7, only self returning walk)
nBridgehead	6	Number of bridgehead atoms
SlogP_VSA6	6	MOE logP VSA Descriptor 6 (0.15 ≤ x < 0.20)
JGI9	6	Nine-ordered mean topological charge
EState_VSA4	6	EState VSA Descriptor 4 (0.72 ≤ x < 1.17)
SRW05	6	Walk count (leg-5, only self returning walk)
ATS0are	6	Moreau-broto autocorrelation of lag 0 weighted by allred-rocow EN
PEOE_VSA7	6	MOE Charge VSA Descriptor 7 (−0.05 ≤ x < 0.00)
Xpc-4dv	5	Four-ordered Chi path-cluster weighted by valence electrons
ZMIC0	5	Zero-ordered Z-modified information content
ATS7m	5	Moreau-broto autocorrelation of lag 7 weighted by mass
NaaO	4	number of aaO

**Table 11:** Toxicity datasets used in experiments.

Dataset	# Molecules	# Inactive	# Active	Ratio of Active Molecules	Molecular Pathway Endpoint
DS1	7044	6743	301	4.3%	Androgen receptor MDA-kb2 AR-luc cell line (NR-AR)
DS2	6572	6349	223	3.4%	Androgen receptor GeneBLAzer AR-UAS-bla-GripTite Cell line (NR-AR-LBD)
DS3	6358	5601	757	11.9%	Aryl hydrocarbon receptor (NR-AhR)
DS4	5661	5368	293	5.2%	Aromatase enzyme (NR-Aromatase)
DS5	6013	5247	766	12.7%	Estrogen receptor α BG1-Luc-4E2 cell line (NR-ER)
DS6	6752	6426	326	4.8%	Estrogen receptor α ER-α-UAS-bla GripTiteTM cell line (NR-ER-LBD)
DS7	6273	6110	163	2.6%	Peroxisome proliferator-activated receptor γ (NR-PPAR-γ)
DS8	5684	4784	900	15.8%	Nuclear factor (erythroid-derived 2)-like 2/antioxidant responsive element (NR-ARE) (SR-ARE)
DS9	6880	6633	247	3.6%	ATAD5 receptor (SR-ATAD5)
DS10	6294	5957	337	5.4%	Heat shock factor response element (SR-HSE)
DS11	5334	4753	881	15.6%	Mitochondrial membrane potential (SR-MMP)
DS12	6586	6191	395	6.0%	p53 signaling pathway (SR-p53)

# molecules denotes the number of drug molecules in the dataset; # inactive and # active, respectively, represent the number of inactive and active drug molecules.

**Table 12:** Modred descriptor information.

Descriptor Name	Number of Descriptors
ABCIndex	2
AcidBase	2
AdjacencyMatrix	13
Aromatic	2
AtomCount	17
Autocorrelation	606
BalabanJ	1
BaryszMatrix	104
BCUT	24
BertzCT	1
BondCount	9
CarbonTypes	11
Chi	56
Constitutional	16
DetourMatrix	14
DistanceMatrix	13
EccentricConnectivityIndex	1
Estate	316
ExtendedTopochemicalAtom	45
FragmentComplexity	1
Framework	1
HydrogenBond	2
InformationContent	42
KappaShapeIndex	3
Lipinski	2
LogS	1
McGowanVolume	1
MoeType	53
MolecularDistanceEdge	19
MolecularId	12
PathCount	21
Polarizability	2
RingCount	138
RotatableBond	2
SLogP	2
TopologicalCharge	21
TopologicalIndex	4
TopoPSA	2
VdwVolumeABC	1
VertexAdjacencyInformation	1
WalkCount	21
Weight	2
WienerIndex	2
ZagrebIndex	4

**Table 13:** Confusion matrix.

	Predicted Positive Class	Predicted Negative Class
Real positive class	*TP* (True positive)	*FN* (False negative)
Real negative class	*FP* (False positive)	*TN* (True negative)

**Table 14:** Default parameter settings of the proposed BACO algorithm and SVM classifier.

Parameter Name	Default Setting
*S*: number of ants in the ant colony	50
*R*: the iteration times of BACO	10
*M*: number of random divisions	40
*ρ*: evaporation factor	0.2
*K*: number of selecting features	20
*ph_ini1_*: initial pheromone concentration in pathway 1	0.4
*ph_ini2_*: initial pheromone concentration in pathway 2	1.0
*ph_min_*: the lower bound of pheromone concentration	0.1
*ph_max_*: the lower bound of pheromone concentration	2.0
*α*, *β*: the weights for three performance metrics	1/3
Kernel function type used in SVM	*rbf*
*σ*: the parameter of kernel function in SVM	5
*C*: the penalty factor in SVM	100

## Data Availability

The Tox21 datasets with SMILES descriptions used in this study can be downloaded from https://pubs.acs.org/doi/abs/10.1021/acs.jcim.0c00908, accessed on 26 September 2024. The Ovarian I and Ovarian II datasets can be downloaded from https://home.ccr.cancer.gov/ncifdaproteomics/ppatterns.asp, accessed on 26 September 2024, and the Colon dataset can be downloaded from https://github.com/sameesayeed007/Feature-Selection-For-High-Dimensional-Imbalanced-Datasets/blob/main/Datasets/colon%202000.xls, accessed on 26 September 2024.

## Supplementary Materials

The following supporting information can be downloaded at: https://www.mdpi.com/article/10.3390/molecules30071548/s1, Table S1: Classification performance of BACO on the DS5~DS8 datasets with different *K* settings; Table S2: Classification performance of BACO on the DS9~DS12 datasets with different *K* settings; Table S3: Classification performance of BACO on the DS5~DS8 datasets with different basic classifiers; Table S4: Classification performance of BACO on the DS9~DS12 datasets with different basic classifiers; Table S5: List of information of the top 20 high-frequency descriptors acquired by BACO on DS2 dataset; Table S6: List of information of the top 20 high-frequency descriptors acquired by BACO on DS3 dataset; Table S7: List of information of the top 20 high-frequency descriptors acquired by BACO on DS4 dataset; Table S8: List of information of the top 20 high-frequency descriptors acquired by BACO on DS5 dataset; Table S9: List of information of the top 20 high-frequency descriptors acquired by BACO on DS6 dataset; Table S10: List of information of the top 20 high-frequency descriptors acquired by BACO on DS8 dataset; Table S11: List of information of the top 20 high-frequency descriptors acquired by BACO on DS9 dataset; Table S12: List of information of the top 20 high-frequency descriptors acquired by BACO on DS10 dataset; Table S13: List of information of the top 20 high-frequency descriptors acquired by BACO on DS11 dataset; Table S14: List of information of the top 20 high-frequency descriptors acquired by BACO on DS12 dataset.

## References

[1] Gupta R., Polaka S., Rajpoot K., Tekade M., Sharma M.C., Tekade R.K. (2022). Importance of toxicity testing in drug discovery and research. Pharmacokinetics and Toxicokinetic Considerations.

[2] Kelleci Çelik F., Karaduman G. (2023). In silico QSAR modeling to predict the safe use of antibiotics during pregnancy. Drug Chem. Toxicol..

[3] Krewski D., Andersen M.E., Tyshenko M.G., Krishnan K., Hartung T., Boekelheide K., Wambaugh J.F., Jones D., Whelan M., Thomas R. (2020). Toxicity testing in the 21st century: Progress in the past decade and future perspectives. Arch. Toxicol..

[4] De P., Kar S., Ambure P., Roy K. (2022). Prediction reliability of QSAR models: An overview of various validation tools. Arch. Toxicol..

[5] Tran T.T.V., Surya Wibowo A., Tayara H., Chong K.T. (2023). Artificial intelligence in drug toxicity prediction: Recent advances, challenges, and future perspectives. J. Chem. Inf. Model..

[6] Achar J., Firman J.W., Tran C., Kim D., Cronin M.T., Öberg G. (2024). Analysis of implicit and explicit uncertainties in QSAR prediction of chemical toxicity: A case study of neurotoxicity. Regul. Toxicol. Pharmacol..

[7] Tropsha A. (2010). Best practices for QSAR model development, validation, and exploitation. Mol. Inform..

[8] Keyvanpour M.R., Shirzad M.B. (2021). An analysis of QSAR research based on machine learning concepts. Curr. Drug Discov. Technol..

[9] Zhang F., Wang Z., Peijnenburg W.J., Vijver M.G. (2023). Machine learning-driven QSAR models for predicting the mixture toxicity of nanoparticles. Environ. Int..

[10] Cerruela García G., Pérez-Parras Toledano J., de Haro García A., García-Pedrajas N. (2019). Filter feature selectors in the development of binary QSAR models. SAR QSAR Environ. Res..

[11] Eklund M., Norinder U., Boyer S., Carlsson L. (2014). Choosing feature selection and learning algorithms in QSAR. J. Chem. Inf. Model..

[12] MotieGhader H., Gharaghani S., Masoudi-Sobhanzadeh Y., Masoudi-Nejad A. (2017). Sequential and mixed genetic algorithm and learning automata (SGALA, MGALA) for feature selection in QSAR. Iran. J. Pharm. Res. IJPR.

[13] Wang Y., Wang B., Jiang J., Guo J., Lai J., Lian X.Y., Wu J. (2021). Multitask CapsNet: An imbalanced data deep learning method for predicting toxicants. ACS Omega.

[14] Idakwo G., Thangapandian S., Luttrell J., Li Y., Wang N., Zhou Z., Hong H., Yang B., Zhang C., Gong P. (2020). Structure–activity relationship-based chemical classification of highly imbalanced Tox21 datasets. J. Cheminform..

[15] Kim C., Jeong J., Choi J. (2022). Effects of Class Imbalance and Data Scarcity on the Performance of Binary Classification Machine Learning Models Developed Based on ToxCast/Tox21 Assay Data. Chem. Res. Toxicol..

[16] Guyon I., Weston J., Barnhill S., Vapnik V. (2002). Gene selection for cancer classification using support vector machines. Mach. Learn..

[17] Dorigo M., Birattari M., Stutzle T. (2006). Ant colony optimization. IEEE Comput. Intell. Mag..

[18] Sun L., Chen Y., Ding W., Xu J. (2024). LEFSA: Label enhancement-based feature selection with adaptive neighborhood via ant colony optimization for multilabel learning. Int. J. Mach. Learn. Cybern..

[19] Yu H., Ni J., Zhao J. (2013). ACOSampling: An ant colony optimization-based undersampling method for classifying imbalanced DNA microarray data. Neurocomputing.

[20] Drucker H., Wu D., Vapnik V.N. (1999). Support vector machines for spam categorization. IEEE Trans. Neural Netw..

[21] Boczar D., Michalska K. (2024). A review of machine learning and QSAR/QSPR Predictions for complexes of organic molecules with cyclodextrins. Molecules.

[22] Rodríguez-Pérez R., Bajorath J. (2022). Evolution of support vector machine and regression modeling in chemoinformatics and drug discovery. J. Comput.-Aided Mol. Des..

[23] Czermiński R., Yasri A., Hartsough D. (2001). Use of support vector machine in pattern classification: Application to QSAR studies. Quant. Struct.-Act. Relatsh..

[24] Du Z., Wang D., Li Y. (2022). Comprehensive evaluation and comparison of machine learning methods in QSAR modeling of antioxidant tripeptides. ACS Omega.

[25] Antelo-Collado A., Carrasco-Velar R., García-Pedrajas N., Cerruela-García G. (2020). Effective feature selection method for class-imbalance datasets applied to chemical toxicity prediction. J. Chem. Inf. Model..

[26] Moriwaki H., Tian Y.S., Kawashita N., Takagi T. (2018). Mordred: A molecular descriptor calculator. J. Cheminform..

[27] Rupapara V., Rustam F., Ishaq A., Lee E., Ashraf I. (2023). Chi-square and PCA based feature selection for diabetes detection with ensemble classifier. Intell. Autom. Soft Comput..

[28] Menze B.H., Kelm B.M., Masuch R., Himmelreich U., Bachert P., Petrich W., Hamprecht F.A. (2009). A comparison of random forest and its Gini importance with standard chemometric methods for the feature selection and classification of spectral data. BMC Bioinform..

[29] Peng H., Long F., Ding C. (2005). Feature selection based on mutual information criteria of max-dependency, max-relevance, and min-redundancy. IEEE Trans. Pattern Anal. Mach. Intell..

[30] Estévez P.A., Tesmer M., Perez C.A., Zurada J.M. (2009). Normalized mutual information feature selection. IEEE Trans. Neural Netw..

[31] Robnik-Šikonja M., Kononenko I. (2003). Theoretical and empirical analysis of ReliefF and RReliefF. Mach. Learn..

[32] Demsar J. (2006). Statistical comparisons of classifiers over multiple data sets. J. Mach. Learn. Res..

[33] Garcia S., Fernandez A., Luengo J., Herrera F. (2010). Advanced nonpara-metric tests for multiple comparisons in the design of experiments in computational intelligence and data mining: Experimental analysis of power. Inf. Sci..

[34] Makarov D.M., Ksenofontov A.A., Budkov Y.A. (2025). Consensus Modeling for Predicting Chemical Binding to Transthyretin as the Winning Solution of the Tox24 Challenge. Chem. Res. Toxicol..

[35] Loh W.Y. (2011). Classification and regression trees. Wiley Data Min. Knowl. Discov..

[36] Nick T.G., Campbell K.M. (2007). Logistic regression. Top. Biostat..

[37] Breiman L. (2001). Random forests. Mach. Learn..

[38] Chen T., Guestrin C. Xgboost: A scalable tree boosting system. Proceedings of the 22nd ACM SIGKDD International Conference on Knowledge Discovery and Data Mining.

[39] Petricoin E.F., Ardekani A.M., Hitt B.A., Levine P.J., Fusaro V.A., Steinberg S.M., Mills G.B., Simone C., Fishman D.A., Kohn E.C. (2002). Use of proteomic patterns in serum to identify ovarian cancer. Lancet.

[40] Alon U., Barkai N., Notterman D.A., Gish K., Ybarra S., Mack D., Levine A.J. (1999). Broad patterns of gene expression revealed by clustering analysis of tumor and normal colon tissues probed by oligonucleotide arrays. Proc. Natl. Acad. Sci. USA.

[41] Srisongkram T. (2023). Ensemble quantitative read-across structure–activity relationship algorithm for predicting skin cytotoxicity. Chem. Res. Toxicol..

[42] Krishnan S.R., Roy A., Gromiha M.M. (2024). Reliable method for predicting the binding affinity of RNA-small molecule interactions using machine learning. Brief. Bioinform..

[43] Clare B.W. (2006). QSAR of aromatic substances: Toxicity of polychlorodibenzofurans. J. Mol. Struct. Theochem.

[44] Podder T., Kumar A., Bhattacharjee A., Ojha P.K. (2023). Exploring regression-based QSTR and i-QSTR modeling for ecotoxicity prediction of diverse pesticides on multiple avian species. Environ. Sci. Adv..

[45] Kumar A., Ojha P.K., Roy K. (2023). QSAR modeling of chronic rat toxicity of diverse organic chemicals. Comput. Toxicol..

[46] Edros R., Feng T.W., Dong R.H. (2023). Utilizing machine learning techniques to predict the blood-brain barrier permeability of compounds detected using LCQTOF-MS in Malaysian Kelulut honey. SAR QSAR Environ. Res..

[47] Fujimoto T., Gotoh H. (2021). Prediction and chemical interpretation of singlet-oxygen-scavenging activity of small molecule compounds by using machine learning. Antioxidants.

[48] Galvez-Llompart M., Hierrezuelo J., Blasco M., Zanni R., Galvez J., de Vicente A., Pérez-García A., Romero D. (2024). Targeting bacterial growth in biofilm conditions: Rational design of novel inhibitors to mitigate clinical and food contamination using QSAR. J. Enzym. Inhib. Med. Chem..

[49] Castillo-Garit J.A., Barigye S.J., Pham-The H., Pérez-Doñate V., Torrens F., Pérez-Giménez F. (2021). Computational identification of chemical compounds with potential anti-Chagas activity using a classification tree. SAR QSAR Environ. Res..

[50] Wang G., Wong K.W., Lu J. (2020). AUC-based extreme learning machines for supervised and semi-supervised imbalanced classification. IEEE Trans. Syst. Man Cybern. Syst..

[51] Saito T., Rehmsmeier M. (2015). The precision-recall plot is more informative than the ROC plot when evaluating binary classifiers on imbalanced datasets. PLoS ONE.

[52] Efron B. (2000). The bootstrap and modern statistics. J. Am. Stat. Assoc..

