# Supplementary material for: Predicting the Toxicity of Drug Molecules with Selecting Effective Descriptors Using a Binary Ant Colony Optimization (BACO) Feature Selection Approach
Source: Molecules. 2025 Mar 31;30(7):1548. doi: 10.3390/molecules30071548 (PMC11990530; doi:10.3390/molecules30071548)
Supplement: Supplementary file 1 [file molecules-30-01548-s001.zip › Table S1.pdf]

Table S1. Classification performance of BACO on DS5~DS8 datasets with different *K* settings.

Number of selected descriptors *K*	F-measure	G-mean	MCC	AUC	PR-AUC
	DS5				
5	0.1896	0.3343	0.2177	0.6342	0.2363
10	0.1988	0.3364	0.2641	0.6709	0.2755
20	0.1997	0.3367	0.2722	0.7198	**0.2948**
30	**0.2024**	**0.3381**	**0.2845**	**0.7438**	0.2886
50	0.1995	0.3354	0.2758	0.7296	0.2891
100	0.2007	0.3358	0.2835	0.7183	0.2735
200	0.1846	0.3203	0.2676	0.6958	0.2360
300	0.1703	0.3064	0.2643	0.6779	0.2187
	DS6				
5	0.1144	0.2447	0.2334	0.6543	0.1425
10	0.1205	0.2507	0.2450	0.6775	0.1660
20	**0.1465**	**0.2801**	0.2488	**0.6854**	0.1732
30	0.1465	0.2801	0.2488	0.6837	**0.1888**
50	0.1442	0.2743	**0.2577**	0.6772	0.1706
100	0.1148	0.2447	0.2391	0.6651	0.1559
200	0.1148	0.2447	0.2391	0.6404	0.1328
300	0.1148	0.2447	0.2391	0.6271	0.1152
	DS7				
5	0.0000	0.0000	0.0000	0.6552	0.0552
10	0.0000	0.0000	0.0000	**0.6731**	0.0538
20	0.0000	0.0000	0.0000	0.6496	0.0547
30	0.0000	0.0000	0.0000	0.6505	**0.0670**
50	0.0000	0.0000	0.0000	0.6418	0.0578
100	0.0000	0.0000	0.0000	0.6337	0.0529
200	0.0000	0.0000	0.0000	0.6351	0.0493
300	0.0000	0.0000	0.0000	0.6305	0.0446
	DS8				
5	0.0586	0.1732	0.0995	0.6734	0.1842
10	0.0862	0.2121	0.1332	0.7820	0.2155
20	0.0884	0.2174	0.1345	0.8159	0.2193
30	0.0902	0.2200	0.1356	0.8269	0.2461
50	**0.0994**	**0.2321**	0.1419	**0.8453**	**0.2778**
100	0.0968	0.2265	**0.1550**	0.8202	0.2336
200	0.0736	0.1964	0.1427	0.7963	0.2198
300	0.0327	0.1284	0.0987	0.7454	0.2073
