# Supplementary material for: Predicting the Toxicity of Drug Molecules with Selecting Effective Descriptors Using a Binary Ant Colony Optimization (BACO) Feature Selection Approach
Source: Molecules. 2025 Mar 31;30(7):1548. doi: 10.3390/molecules30071548 (PMC11990530; doi:10.3390/molecules30071548)
Supplement: Supplementary file 1 [file molecules-30-01548-s001.zip › Table S10.pdf]

Table S10. List of information about the top 20 high-frequency descriptors acquired by BACO on the DS8 dataset.

Descriptor Name	Frequency	Descriptor Definition
nG12FaRing	13	12-or-greater-membered aromatic fused ring count
NdssS	12	number of dssS
nG12FRing	8	12-or-greater-membered fused ring count
SRW09	8	walk count (leg-9, only self returning walk)
Lipinski	8	Lipinski rule of five
ATS5pe	7	moreau-broto autocorrelation of lag 5 weighted by pauling EN
Xch-3d	7	3-ordered Chi chain weighted by sigma electrons
SlogP_VSA8	6	MOE logP VSA Descriptor 8 (0.25 <= x < 0.30)
PEOE_VSA9	5	MOE Charge VSA Descriptor 9 (0.05 <= x < 0.10)
SaaaC	5	sum of aaaC
C3SP2	5	SP2 carbon bound to 3 other carbons
nFaHRing	5	aromatic fused hetero ring count
SddC	4	sum of ddC
SdssC	4	sum of dssC
ATS7pe	4	moreau-broto autocorrelation of lag 7 weighted by pauling EN
SlogP_VSA2	4	MOE logP VSA Descriptor 2 (-0.40 <= x < -0.20)
Si	4	sum of constitutional weighted by ionization potential
n5aRing	4	5-membered aromatic ring count
n8FRing	4	8-membered fused ring count
MIC1	4	1-ordered modified information content
