# Supplementary material for: Predicting the Toxicity of Drug Molecules with Selecting Effective Descriptors Using a Binary Ant Colony Optimization (BACO) Feature Selection Approach
Source: Molecules. 2025 Mar 31;30(7):1548. doi: 10.3390/molecules30071548 (PMC11990530; doi:10.3390/molecules30071548)
Supplement: Supplementary file 1 [file molecules-30-01548-s001.zip › Table S11.pdf]

**Table S11.** List of information about the top 20 high-frequency descriptors acquired by BACO on the DS9 dataset.

| Descriptor Name | Frequency | Descriptor Definition |
|---|---|---|
| n9HRing | 9 | 9-membered hetero ring count |
| n9Ring | 9 | 9-membered ring count |
| LabuteASA | 7 | Labute’s Approximate Surface Area |
| JGI9 | 7 | 9-ordered mean topological charge |
| EState_VSA2 | 7 | EState VSA Descriptor 2 ( -0.39 <= x < 0.29) |
| ATS1dv | 7 | moreau-broto autocorrelation of lag 1 weighted by valence electrons |
| ATS7d | 7 | moreau-broto autocorrelation of lag 7 weighted by sigma electrons |
| ATSC4pe | 7 | centered moreau-broto autocorrelation of lag 4 weighted by pauling EN |
| EState_VSA8 | 7 | EState VSA Descriptor 8 ( 2.05 <= x < 4.69) |
| Xc-5d | 6 | 5-ordered Chi cluster weighted by sigma electrons |
| n4HRing | 6 | 4-membered hetero ring count |
| n9ARing | 6 | 9-membered aliphatic ring count |
| nG12AHRing | 6 | 12-or-greater-membered aliphatic hetero ring count |
| nFARing | 6 | aliphatic fused ring count |
| MWC06 | 6 | walk count (leg-6) |
| MWC08 | 6 | walk count (leg-8) |
| IC2 | 6 | 2-ordered neighborhood information content |
| nBr | 6 | number of Br atoms |
| PEOE_VSA5 | 6 | MOE Charge VSA Descriptor 5 (-0.15 <= x < -0.10) |
| AATS0dv | 6 | averaged moreau-broto autocorrelation of lag 0 weighted by valence electrons |
