# Supplementary material for: Predicting the Toxicity of Drug Molecules with Selecting Effective Descriptors Using a Binary Ant Colony Optimization (BACO) Feature Selection Approach
Source: Molecules. 2025 Mar 31;30(7):1548. doi: 10.3390/molecules30071548 (PMC11990530; doi:10.3390/molecules30071548)
Supplement: Supplementary file 1 [file molecules-30-01548-s001.zip › Table S12.pdf]

Table S12. List of information about the top 20 high-frequency descriptors acquired by BACO on the DS10 dataset.

Descriptor Name	Frequency	Descriptor Definition
SdS	13	sum of dS
NdS	11	number of dS
SsssN	11	number of ssssN
NdsssP	8	number of dsssP
nO	7	number of O atoms
ATSC5pe	7	centered moreau-broto autocorrelation of lag 5 weighted by pauling EN
ATSC3dv	7	centered moreau-broto autocorrelation of lag 3 weighted by valence electrons
ZMIC3	7	3-ordered Z-modified information content
SMR_VSA2	7	MOE MR VSA Descriptor 2 (1.29 <= x < 1.82)
ZMIC0	7	0-ordered Z-modified information content
SMR_VSA6	7	MOE MR VSA Descriptor 6 (2.75 <= x < 3.05)
nH	6	number of H atoms
Mi	6	mean of constitutional weighted by ionization potential
NaasC	6	number of aasC
nS	6	number of S atoms
n9FARing	6	9-membered aliphatic fused ring count
MPC9	6	9-ordered path count
NssO	6	number of ssO
NsNH2	6	number of sNH2
VSA_EState2	6	VSA EState Descriptor 2 (4.78 <= x < 5.00)
