# Supplementary material for: Predicting the Toxicity of Drug Molecules with Selecting Effective Descriptors Using a Binary Ant Colony Optimization (BACO) Feature Selection Approach
Source: Molecules. 2025 Mar 31;30(7):1548. doi: 10.3390/molecules30071548 (PMC11990530; doi:10.3390/molecules30071548)
Supplement: Supplementary file 1 [file molecules-30-01548-s001.zip › Table S13.pdf]

Table S13. List of information about the top 20 high-frequency descriptors acquired by BACO on the DS11 dataset.

Descriptor Name	Frequency	Descriptor Definition
Lipinski	14	Lipinski rule of five
NddsN	11	number of ddsN
NaasC	10	number of aasC
SaaN	8	sum of aaN
FCSP3	8	the fraction of C atoms that are SP3 hybridized
SMR_VSA9	7	MOE MR VSA Descriptor 9 (3.80 <= x < 4.00)
fMF	7	molecular framework ratio
n6aRing	6	6-membered aromatic ring count
SMR_VSA3	6	MOE MR VSA Descriptor 3 (1.82 <= x < 2.24)
WPath	6	Wiener index
nAcid	6	acidic group count
n8FARing	5	8-membered aliphatic fused ring count
n6Ring	5	6-membered ring count
SlogP_VSA11	5	MOE logP VSA Descriptor 11 (0.50 <= x < 0.60)
ZMIC1	5	1-ordered Z-modified information content
nG12FaRing	5	12-or-greater-membered aromatic fused ring count
nAromBond	5	aromatic bonds count
ATS4m	5	moreau-broto autocorrelation of lag 4 weighted by mass
ATS2dv	5	moreau-broto autocorrelation of lag 2 weighted by valence electrons
nFRing	5	fused ring count
