# Supplementary material for: Predicting the Toxicity of Drug Molecules with Selecting Effective Descriptors Using a Binary Ant Colony Optimization (BACO) Feature Selection Approach
Source: Molecules. 2025 Mar 31;30(7):1548. doi: 10.3390/molecules30071548 (PMC11990530; doi:10.3390/molecules30071548)
Supplement: Supplementary file 1 [file molecules-30-01548-s001.zip › Table S14.pdf]

Table S14. List of information about the top 20 high-frequency descriptors acquired by BACO on the DS12 dataset.

Descriptor Name	Frequency	Descriptor Definition
n9FRing	6	9-membered fused ring count
MPC5	6	5-ordered path count
nBondsD	6	number of double bonds in non-kekulized structure
nX	6	number of halogen atoms
nG12FARing	5	12-or-greater-membered aliphatic fused ring count
TopoPSA(NO)	5	topological polar surface area (use only nitrogen and oxygen)
SaaaC	5	sum of aaaC
fragCpx	5	fragment complexity
ATS6pe	5	moreau-broto autocorrelation of lag 6 weighted by pauling EN
EState_VSA2	5	EState VSA Descriptor 2 (-0.39 <= x < 0.29)
Zagreb1	5	Zagreb index (version 1)
ATS2m	5	moreau-broto autocorrelation of lag 2 weighted by mass
ATSC1d	5	centered moreau-broto autocorrelation of lag 1 weighted by sigma electrons
nFARing	5	aliphatic fused ring count
nG12FaRing	5	12-or-greater-membered aromatic fused ring count
n3Ring	5	3-membered ring count
AATS0m	5	averaged moreau-broto autocorrelation of lag 0 weighted by mass
ATS1are	4	moreau-broto autocorrelation of lag 1 weighted by allred-rocow EN
MIC0	4	0-ordered modified information content
EState_VSA8	4	EState VSA Descriptor 8 (2.05 <= x < 4.69)
