# Supplementary material for: Predicting the Toxicity of Drug Molecules with Selecting Effective Descriptors Using a Binary Ant Colony Optimization (BACO) Feature Selection Approach
Source: Molecules. 2025 Mar 31;30(7):1548. doi: 10.3390/molecules30071548 (PMC11990530; doi:10.3390/molecules30071548)
Supplement: Supplementary file 1 [file molecules-30-01548-s001.zip › Table S2.pdf]

Table S2. Classification performance of BACO on DS9~DS12 datasets with different *K* settings.

**Number of selected descriptors *K***	**F-measure**	**G-mean**	**MCC**	**AUC**	**PR-AUC**
	DS9				
5	0.0172	0.0596	0.0493	0.5736	0.0796
10	**0.0236**	**0.0848**	**0.0833**	**0.6434**	0.1250
20	**0.0236**	**0.0848**	**0.0833**	**0.6434**	0.1490
30	0.0235	**0.0848**	0.0739	0.6418	**0.1507**
50	0.0235	**0.0848**	0.0739	0.6418	0.1442
100	0.0235	**0.0848**	0.0739	0.6418	0.1247
200	0.0089	0.0302	0.0297	0.5979	0.1120
300	0.0089	0.0302	0.0297	0.5979	0.1078
	DS10				
5	0.0303	0.0960	0.0850	0.7029	0.0991
10	0.0211	0.0650	0.0636	0.7195	0.1127
20	**0.0311**	**0.0970**	**0.0947**	0.7225	0.1229
30	**0.0311**	**0.0970**	**0.0947**	**0.7454**	**0.1559**
50	0.0286	0.0918	0.0892	0.6938	0.1230
100	0.0170	0.0588	0.0572	0.6753	0.0939
200	0.0000	0.0000	0.0000	0.6431	0.0881
300	0.0000	0.0000	0.0000	0.6409	0.0725
	DS11				
5	0.1289	0.2541	0.1483	0.8026	0.2996
10	0.1881	0.3283	0.2115	0.8278	0.3515
20	0.2722	0.4110	0.2816	0.8471	0.3845
30	0.2974	0.4338	0.3082	0.8544	0.4262
50	0.3711	0.4963	0.3724	0.8691	**0.4460**
100	**0.4152**	**0.5297**	**0.4158**	**0.8930**	0.4351
200	0.4074	0.5227	0.4119	0.8875	0.4308
300	0.3810	0.4994	0.3948	0.8702	0.4276
	DS12				
5	0.0412	0.1120	0.0658	0.5976	0.0977
10	0.0524	0.1609	0.1074	0.6507	0.1444
20	0.0620	0.1778	0.1142	0.6778	0.1871
30	**0.0794**	**0.2029**	**0.1414**	**0.6981**	0.1982
50	0.0665	0.1831	0.1280	0.6701	**0.2116**
100	0.0496	0.1557	0.1287	0.6456	0.1879
200	0.0153	0.0553	0.0461	0.5949	0.1074
300	0.0053	0.0232	0.0226	0.5933	0.0818
