# Supplementary material for: Predicting the Toxicity of Drug Molecules with Selecting Effective Descriptors Using a Binary Ant Colony Optimization (BACO) Feature Selection Approach
Source: Molecules. 2025 Mar 31;30(7):1548. doi: 10.3390/molecules30071548 (PMC11990530; doi:10.3390/molecules30071548)
Supplement: Supplementary file 1 [file molecules-30-01548-s001.zip › Table S3.pdf]

Table S3. Classification performance of BACO on DS5~DS8 datasets with different basic classifiers.

Classifier	F-measure	G-mean	MCC	AUC	PR-AUC
			DS5		
SVM	0.1997	**0.3367**	0.2722	0.7198	**0.2948**
CART	0.1676	0.2978	0.2571	0.7316	0.2237
LR	0.1991	0.3134	0.2497	0.6849	0.2924
RF	**0.2032**	0.3259	0.2680	0.7651	0.2650
xgboost	0.1997	0.3306	**0.2835**	**0.7984**	0.2542
			DS6		
SVM	0.1465	0.2801	0.2488	**0.6854**	0.1732
CART	0.1153	0.2475	0.2038	0.6472	0.1342
LR	**0.1492**	0.2597	0.2215	0.6359	0.1628
RF	0.1308	**0.2810**	0.2401	0.6617	**0.1759**
xgboost	0.1277	0.2644	**0.2691**	0.6682	0.1603
			DS7		
SVM	0.0000	0.0000	0.0000	0.6496	0.0547
CART	0.0271	0.0144	0.0333	0.6699	0.0654
LR	0.0000	0.0000	0.0000	0.6190	0.0478
RF	0.0596	**0.0638**	0.0547	0.6938	**0.0792**
xgboost	**0.0887**	0.0552	**0.0786**	**0.7152**	0.0681
			DS8		
SVM	**0.0884**	**0.2174**	0.1345	**0.8159**	0.2193
CART	0.0698	0.1792	0.1189	0.7702	0.1886
LR	0.0824	0.2095	**0.1373**	0.8026	0.2174
RF	0.0739	0.1954	0.1359	0.7874	**0.2266**
xgboost	0.0796	0.2040	0.1336	0.7965	0.2182
