# Supplementary material for: Predicting the Toxicity of Drug Molecules with Selecting Effective Descriptors Using a Binary Ant Colony Optimization (BACO) Feature Selection Approach
Source: Molecules. 2025 Mar 31;30(7):1548. doi: 10.3390/molecules30071548 (PMC11990530; doi:10.3390/molecules30071548)
Supplement: Supplementary file 1 [file molecules-30-01548-s001.zip › Table S4.pdf]

Table S4. Classification performance of BACO on DS9~DS12 datasets with different basic classifiers.

Classifier	F-measure	G-mean	MCC	AUC	PR-AUC
			DS9		
SVM	0.0236	0.0848	**0.0833**	0.6434	**0.1490**
CART	0.0336	0.1122	0.0750	0.6032	0.1240
LR	0.0245	0.0808	0.0758	**0.6891**	0.1333
RF	**0.0477**	0.1299	0.0802	0.6344	0.1376
xgboost	0.0430	**0.1341**	0.0777	0.6571	0.1475
			DS10		
SVM	0.0311	0.0970	0.0947	0.7225	**0.1229**
CART	0.0528	0.1125	0.1020	0.7408	0.1147
LR	0.0732	0.1070	0.0996	0.7343	0.1210
RF	0.0630	0.1115	**0.1221**	**0.7572**	0.1195
xgboost	**0.0821**	**0.1292**	0.1172	0.7508	0.1176
			DS11		
SVM	**0.2722**	0.4110	**0.2816**	**0.8471**	0.3845
CART	0.2448	0.4070	0.2635	0.8295	0.3796
LR	0.2589	0.4188	0.2757	0.8176	0.3757
RF	0.2697	**0.4242**	0.2748	0.8304	0.4102
xgboost	0.2702	0.4156	0.2722	0.8444	**0.4244**
			DS12		
SVM	**0.0620**	**0.1778**	0.1142	0.6778	**0.1871**
CART	0.0604	0.1633	0.1032	0.6518	0.1646
LR	0.0595	0.1658	0.1112	0.6634	0.1533
RF	0.0578	0.1742	**0.1201**	0.6797	0.1679
xgboost	0.0526	0.1666	0.1074	**0.6905**	0.1754
