# Supplementary material for: Predicting the Toxicity of Drug Molecules with Selecting Effective Descriptors Using a Binary Ant Colony Optimization (BACO) Feature Selection Approach
Source: Molecules. 2025 Mar 31;30(7):1548. doi: 10.3390/molecules30071548 (PMC11990530; doi:10.3390/molecules30071548)
Supplement: Supplementary file 1 [file molecules-30-01548-s001.zip › Table S5.pdf]

Table S5. List of information about the top 20 high-frequency descriptors acquired by BACO on the DS2 dataset.

Descriptor Name	Frequency	Descriptor Definition
nG12FARing	16	12-or-greater-membered aliphatic fused ring count
nG12FRing	13	12-or-greater-membered fused ring count
nFAHRing	10	aliphatic fused hetero ring count
nFHRing	9	fused hetero ring count
n6ARing	8	6-membered aliphatic ring count
nG12FHRing	8	12-or-greater-membered fused hetero ring count
SRW05	7	walk count (leg-5, only self returning walk)
n5HRing	6	5-membered hetero ring count
n5ARing	5	5-membered aliphatic ring count
nG12FAHRing	5	12-or-greater-membered aromatic fused hetero ring count
nBridgehead	5	number of bridgehead atoms
AATSC0Z	4	averaged and centered moreau-broto autocorrelation of lag 0 weighted by atomic number
SssNH	4	sum of ssNH
PEOE_VSA11	4	MOE Charge VSA Descriptor 11 (0.15 <= x < 0.20)
SMR_VSA4	4	MOE MR VSA Descriptor 4 (2.24 <= x < 2.45)
nG12aHRing	4	12-or-greater-membered aromatic hetero ring count
n11ARing	4	11-membered aromatic ring count
JGI6	4	6-ordered mean topological charge
Xc-6dv	4	6-ordered Chi cluster weighted by valence electrons
StCH	4	sum of tCH
