# Supplementary material for: Predicting the Toxicity of Drug Molecules with Selecting Effective Descriptors Using a Binary Ant Colony Optimization (BACO) Feature Selection Approach
Source: Molecules. 2025 Mar 31;30(7):1548. doi: 10.3390/molecules30071548 (PMC11990530; doi:10.3390/molecules30071548)
Supplement: Supplementary file 1 [file molecules-30-01548-s001.zip › Table S6.pdf]

Table S6. List of information about the top 20 high-frequency descriptors acquired by BACO on the DS3 dataset.

Descriptor Name	Frequency	Descriptor Definition
nG12FaRing	18	12-or-greater-membered aromatic fused ring count
nFaRing	13	aromatic fused ring count
nG12FRing	6	12-or-greater-membered fused ring count
NaaaC	6	number of aaaC
NaasN	6	number of aasN
NsCl	6	number of sCl
ATS5dv	5	moreau-broto autocorrelation of lag 5 weighted by valence electrons
JGI4	5	4-ordered mean topological charge
n11Ring	5	11-membered ring count
nBonds	5	number of all bonds in non-kekulized structure
ATS4v	5	moreau-broto autocorrelation of lag 4 weighted by vdw volume
ATSC0pe	5	centered moreau-broto autocorrelation of lag 0 weighted by pauling EN
SdS	5	sum of dS
PEOE_VSA3	5	MOE Charge VSA Descriptor 3 (-0.25 <= x < -0.20)
ATS4d	4	moreau-broto autocorrelation of lag 4 weighted by valence electrons
ATS8Z	4	moreau-broto autocorrelation of lag 8 weighted by atomic number
ZMIC5	4	5-ordered Z-modified information content
n5AHRing	4	5-membered aromatic hetero ring count
NsssP	4	number of sssP
SsF	4	sum of sF
