# Supplementary material for: Predicting the Toxicity of Drug Molecules with Selecting Effective Descriptors Using a Binary Ant Colony Optimization (BACO) Feature Selection Approach
Source: Molecules. 2025 Mar 31;30(7):1548. doi: 10.3390/molecules30071548 (PMC11990530; doi:10.3390/molecules30071548)
Supplement: Supplementary file 1 [file molecules-30-01548-s001.zip › Table S7.pdf]

Table S7. List of information about the top 20 high-frequency descriptors acquired by BACO on the DS4 dataset.

Descriptor Name	Frequency	Descriptor Definition
SaasN	29	sum of aasN
EState_VSA5	8	EState VSA Descriptor 5 (1.17 <= x < 1.54)
NaasN	8	number of aasN
fMF	8	molecular framework ratio
NdsssP	7	number of dsssP
ATS6se	7	moreau-broto autocorrelation of lag 6 weighted by sanderson EN
ZMIC2	6	2-ordered Z-modified information content
PEOE_VSA6	6	MOE Charge VSA Descriptor 6 (-0.10 <= x < -0.05)
n5aHRing	6	5-membered aromatic hetero ring count
ATS1v	6	moreau-broto autocorrelation of lag 1 weighted by vdw volume
TIC4	6	4-ordered neighborhood total information content
SlogP_VSA7	6	MOE logP VSA Descriptor 7 (0.20 <= x < 0.25)
CIC2	6	2-ordered complementary information content
LabuteASA	6	Labute’s Approximate Surface Area
ATS3dv	5	moreau-broto autocorrelation of lag 3 weighted by valence electrons
n10HRing	5	10-membered hetero ring count
nS	5	number of S atoms
SaaN	5	sum of aaN
n7FARing	5	7-membered aliphatic fused ring count
SZ	5	sum of constitutional weighted by atomic number
