# Supplementary material for: Predicting the Toxicity of Drug Molecules with Selecting Effective Descriptors Using a Binary Ant Colony Optimization (BACO) Feature Selection Approach
Source: Molecules. 2025 Mar 31;30(7):1548. doi: 10.3390/molecules30071548 (PMC11990530; doi:10.3390/molecules30071548)
Supplement: Supplementary file 1 [file molecules-30-01548-s001.zip › Table S8.pdf]

Table S8. List of information about the top 20 high-frequency descriptors acquired by BACO on the DS5 dataset.

Descriptor Name	Frequency	Descriptor Definition
nG12FARing	20	12-or-greater-membered aliphatic fused ring count
nG12FRing	18	12-or-greater-membered fused ring count
nG12FAHRing	11	12-or-greater-membered aliphatic fused hetero ring count
nG12FHRing	8	12-or-greater-membered fused hetero ring count
nFAHRing	8	aliphatic fused hetero ring count
C3SP3	7	SP3 carbon bound to 3 other carbons
nRing	5	ring count
MPC3	5	3-ordered path count
WPol	5	Wiener polarity index
n5AHRing	5	5-membered aliphatic hetero ring count
PEOE_VSA2	5	MOE Charge VSA Descriptor 2 (-0.30 <= x < -0.25)
C2SP3	5	SP3 carbon bound to 2 other carbons
SRW09	5	walk count (leg-9, only self returning walk)
ATS7pe	5	moreau-broto autocorrelation of lag 7 weighted by pauling EN
SlogP_VSA10	5	MOE logP VSA Descriptor 10 (0.40 <= x < 0.50)
nHeavyAtom	4	number of heavy atoms
n7Ring	4	7-membered ring count
VMcGowan	4	McGowan volume
JGI5	4	5-ordered mean topological charge
ATS2v	4	moreau-broto autocorrelation of lag 2 weighted by vdw volume
