# Supplementary material for: Predicting the Toxicity of Drug Molecules with Selecting Effective Descriptors Using a Binary Ant Colony Optimization (BACO) Feature Selection Approach
Source: Molecules. 2025 Mar 31;30(7):1548. doi: 10.3390/molecules30071548 (PMC11990530; doi:10.3390/molecules30071548)
Supplement: Supplementary file 1 [file molecules-30-01548-s001.zip › Table S9.pdf]

Table S9. List of information about the top 20 high-frequency descriptors acquired by BACO on the DS6 dataset.

Descriptor Name	Frequency	Descriptor Definition
StCH	24	sum of tCH
NtCH	12	number of tCH
nG12FRing	6	12-or-greater-membered fused ring count
Sse	6	sum of constitutional weighted by sanderson EN
AATSC0m	5	averaged and centered moreau-broto autocorrelation of lag 0 weighted by mass
n10FaRing	5	10-membered aromatic fused ring count
ATS5i	5	moreau-broto autocorrelation of lag 5 weighted by ionization potential
nARing	5	aliphatic ring count
n8AHRing	5	8-membered aliphatic hetero ring count
JGI9	5	9-ordered mean topological charge
SlogP_VSA6	5	MOE logP VSA Descriptor 6 (0.15 <= x < 0.20)
SMR	5	Wildman-Crippen MR
Xp-7d	5	7-ordered Chi path weighted by sigma electrons
ATS8d	5	moreau-broto autocorrelation of lag 8 weighted by sigma electrons
ATS5p	5	moreau-broto autocorrelation of lag 5 weighted by polarizability
SZ	5	sum of constitutional weighted by atomic number
SMR_VSA2	5	MOE MR VSA Descriptor 2 (1.29 <= x < 1.82)
n6FRing	5	6-membered fused ring count
nI	4	number of I atoms
ATS2p	4	moreau-broto autocorrelation of lag 2 weighted by polarizability
